# The Effect of Antipsychotics and Their Combinations with Other Psychotropic Drugs on Electrocardiogram Intervals Other Than QTc among Jordanian Adult Outpatients

**DOI:** 10.3390/biomedicines11010013

**Published:** 2022-12-22

**Authors:** Nailya Bulatova, Noor Altaher, Radwan BaniMustafa, Akram Al-Saleh, Haya Yasin, Mohammed Zawiah, Hala Khalefah, Mokhtar Ghilan, Ala’a Al-Lahham, Mohummad Hudaib, Batoul AlKhawaldeh, Mahmoud Nasr

**Affiliations:** 1 Department of Biopharmaceutics and Clinical Pharmacy, School of Pharmacy, The University of Jordan, Amman 11942, Jordan; 2 Department of Internal Medicine, School of Medicine, The University of Jordan, Amman 11942, Jordan

**Keywords:** antipsychotics, ECG, psychotropics combinations, Jordanians, polytherapy

## Abstract

The ECG changes produced by antipsychotics and other psychotropic medications are studied mostly regarding QTc interval prolongation. This study aimed to investigate ECG changes beyond long QTc interval produced by psychotropic medications. A cross-sectional study was conducted to assess the effect of these agents on RR, PR, TpTe intervals and TpTe/QT ratio among Jordanian outpatients. The RR interval was significantly shorter among patients on TCAs versus those not receiving TCAs and among patients on polytherapy versus those on monotherapy (*p* < 0.05 for both comparisons), when adjusted for age, gender, BMI, caffeine intake, smoking, presence of diabetes mellitus, cardiovascular disease and medications known to produce heart rate changes. Positive correlations were found between the PR interval and age in patients treated with SGAs, SSRIs, citalopram, polytherapy and in the total sample (*p* < 0.01 for all). Inverse correlations were found between the RR interval and the number of psychotropic medications among patients treated with SSRIs and in the whole study sample (*p* < 0.01 for both). In conclusion, various ECG changes beyond QTc interval prolongation are observed in patients on antipsychotics and other psychotropic medications, in those on polytherapy. It is recommended to obtain an ECG before starting patients on psychotropic drugs known to produce electrocardiographic changes and their combinations.

## 1. Introduction

Patients with mental disorders who receive antipsychotics and other psychotropic medications have higher mortality compared to the general population [1]. Sudden cardiac death (SCD) accounted for 15–40% of sudden unexpected deaths in schizophrenia while around 10% of SCD may be due to arrhythmias from cardiac electric defects that can be detected by electrocardiogram (ECG) [2]. Several antipsychotics have been shown to produce ECG changes and the most investigated is prolongation of QTc interval [3,4], which is known to predict a polymorphic ventricular tachyarrhythmia, torsade de points (TdP) that, in turn, may result in sudden cardiac death (SCD) [5].

The ECG changes other than long QTc interval (LQTc) produced by antipsychotics and other psychotropic agents, are much less studied. They include changes in duration of QRS interval (which reflects the depolarization of the ventricles) [6] and PR interval (the period between the onset of atrial depolarization and the onset of ventricular depolarization) [7], heart rate [8,9] which is inversely associated with the RR interval, morphological changes of P and T waves [2,10], axis deviation, pathological Q wave [2,11] and ST-segment abnormality [2]. Among patients with schizophrenia who were switched from olanzapine to risperidone, a significant decrease in PR interval, but no change in RR interval were observed [7]. In another study, schizophrenia patients developed a decrease in low to high frequency power, an index of heart rate variability, after six weeks of risperidone treatment [8]. A comparison of P-wave duration and P-wave dispersion (measures of proneness to atrial fibrillation) between schizophrenia patients and healthy volunteers after parenteral zisperidone administration showed that the initial P-wave dispersion was significantly longer in patients than in healthy controls [9]. In 37 patients switched to sertindole, prominent T-wave morphology changes occurred, sometimes without concomitant prolongation of the QTcF interval [10]. Among 4486 Danish schizophrenia patients, ECG characteristics were investigated in association with psychotropic drugs, where patients with schizophrenia demonstrated higher median heart rate and pathological Q waves than controls. Among patients with schizophrenia only, redeemed prescriptions of antipsychotics (most notably, clozapine and with the exception of aripiprazole), antipsychotic polypharmacy and benzodiazepines were associated with abnormal ECGs. On the other hand, redeemed prescriptions of antidepressants, with the exception of selective serotonin reuptake inhibitors (SSRIs), selective norepinephrine reuptake inhibitors (SNRIs) and lithium, were not associated with differential risk of abnormal ECGs [11]. In the chart audit of 169 patients with schizophrenia and schizoaffective disorder in a long-stay inpatient unit, more than half (52.1%) had at least one ECG abnormality other than long QTc interval, and one-fifth (20.7%) had two or more ECG abnormalities [2].

TpTe interval and TpTe/QT ratio are being used increasingly in the assessment of arrhythmogenicity. TpTe is the time interval measured from the peak of the T wave to the end of the T wave, which coincides with the repolarization time of epicardium cells until the completion of myocardial M cell repolarization. Prolonged TpTe interval is associated with increased transmual dispersion of repolarization (TDR), which, in turn, is associated with the occurrence of ventricular tachycardia (VT) [12]. We found two studies that investigated the effect of antipsychotics on the TpTe interval and TpTe/QT ratio. In children with attention deficit disorder, the TpTe and TpTe/QT ratio were significantly higher in the risperidone group compared to that of the untreated group [13]. In a randomized double-blind study in schizophrenia patients, treatment with sertindole was associated with an increase in TpTe, along with other ECG changes, while quetiapine caused no increase in TpTe compared to baseline. Although quetiapine (400–600 mg) did not show worsening of repolarization measures, some individual patients did experience significant worsening of repolarization [14]. Studies related to TpTe interval and TpTe ratio changes produced by antidepressants are confined to one study of amitriptyline poisoning in pigs, which found no changes in these parameters [15].

A very few studies investigated the effect of antipsychotics’ combinations on the ECG, most commonly on the QTc interval [1,16,17], including a recent publication by our group [18]. A recent study reported ECG abnormalities in relation to psychotropic polypharmacy in schizophrenia and schizoaffective disorders [2].

In order to address the knowledge gap in this area, this study aimed to investigate ECG changes other than LQTc (duration of RR, PR and TpTe intervals and TpTe/QT ratio) in Jordanian patients treated with antipsychotics and their combinations.

## 2. Materials and Methods

This is a part of the cross-sectional study that investigated the prevalence of LQTc among Jordanian patients treated with psychotropic drugs. The study was conducted in the outpatient psychiatric clinics at Jordan University Hospital (JUH) from March 2018 till March 2019. The study received ethical approval from the Institutional Review Board (IRB) of the JUH. The part of the study that reported QTc interval changes was recently published and contains further methodology details [18]. A surface ECG was recorded in a supine position after a 5-min rest using a 12-lead Mortara ELI 230 portable ECG recorder (25 mm/s paper speed, 10 mm/mV amplitude). The ECG records were analyzed by a fellow in cardiology who was blind to the patients’ condition, study hypothesis, and treatment status and was not involved in the patients’ care.

ECG parameters measured:

- RR interval
- PR interval
- TpTe interval [the interval between the peak and the end of the T wave (Tpeak to Tend)] [19]
- TpTe interval divided by QT interval
- Morphological changes of T wave
- Fragmented QRS

All measurements were conducted in the standard lead II.

All statistical analyses were performed using SPSS version 22 (IBM Corp.; Armonk, NY, USA). The Kolmogorov-Smirnov and Shapiro-Wilk tests were conducted for the normality of data distribution. Frequencies and mean values with standard deviations (SD) were calculated. For the comparison of continuous variables between two treatment groups, Quade’s test (a non-parametric ANCOVA) was performed with the following confounding factors: age, gender, BMI, smoking, caffeine intake, presence of diabetes mellitus, cardiovascular disease and medications known to produce either bradycardia (beta-blockers or calcium channel blockers) or tachycardia (beta_2_-adrenergic agonists or thyroid hormones). To assess the differences in continuous variables between multiple groups for non-normally distributed data, the Kruskal-Wallis test was conducted and followed by the Mann-Whitney U test. Pearson correlation was conducted to assess relationship of different ECG parameters with age and the number of psychotropic medications.

*p*-values < 0.05 were considered statistically significant.

## 3. Results

### 3.1. Patient Demographic and Clinical Data

Three hundred and seven patients were included in the study; the mean age was 36.3 ± 13.5 years; 140 (45.6%) patients were males and 167 (54.4%) were females (Table 1). The mean BMI was 26.7 ± 5.1; 36.8% of patients were smokers and 75.9% admitted caffeine intake.

Anxiety disorder was the most common psychiatric condition among study participants (41.4%), followed by major depressive disorder (28.7%), bipolar disorder (17.3%) and schizophrenia (4.9%). The most common nonpsychiatric conditions were hypertension (8.4%) and diabetes mellitus (5.6%). Medications known to produce tachycardia (beta_2_ adrenergic agonists and thyroid hormones) were prescribed to 7 (2.3%) patients, and 12 (3.9%) patients were on medications known to produce bradycardia (beta-blockers and calcium channel blockers).

### 3.2. Description of Psychotropic Medications

**Figure 1:**
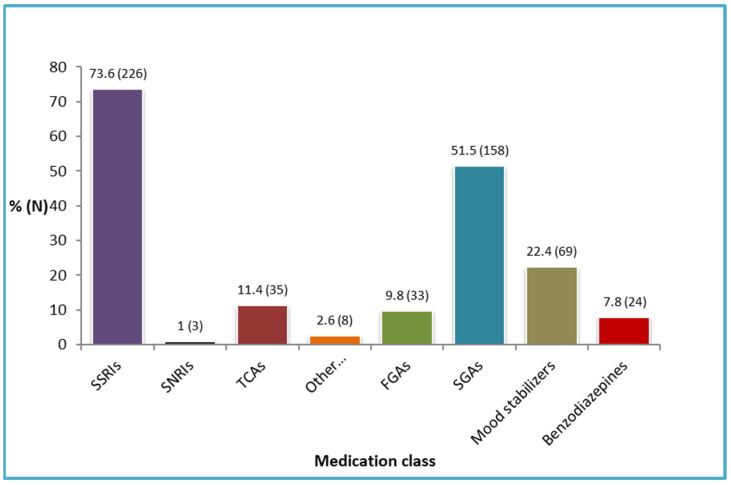
Frequency of psychotropic medications classes (*n* = 307).

The most commonly prescribed psychotropic medications were selective serotonin reuptake inhibitors (SSRIs) (73.4%) and second-generation antipsychotics (SGAs) (51.5%), followed by mood stabilizers (22.4%), tricyclic antidepressants (TCAs) (11.4%), and first-generation antipsychotics (FGAs) (9.8%) (Figure 1).

As for individual psychotropic drugs, the most commonly prescribed among SSRIs were citalopram and fluoxetine (52.2% and 28.2%, respectively), among TCAs were melitracen and amitriptyline (51.4% and 37.1%, respectively) among FGAs was flupentixol (78.8%), among SGAs were quetiapine and olanzapine (46.2% and 27.9%, respectively), among mood stabilizers were valproate and lamotrigine (39.1% and 30.4%, respectively) and among benzodiazepines was bromazepam (70.8%) (Table 2).

**Figure 2:**
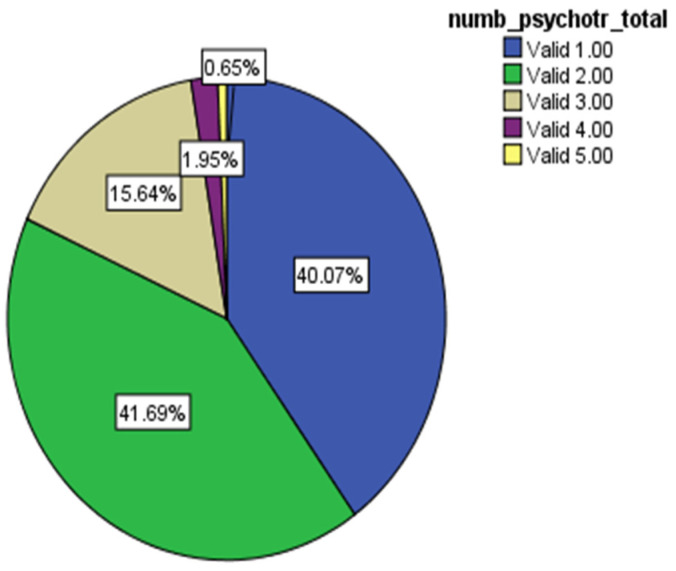
Number of psychotropic medications prescribed.

More than half of patients (*n* = 184; 59.9%) received combined psychotropic therapy. The mean number of psychotropic medications was 1.81 ± 0.81, ranging between 1 and 5. A large proportion of patients received two (*n* = 128; 41.7%) and 48 patients (15.6%) received three psychotropic medications (Figure 2).

**Figure 3:**
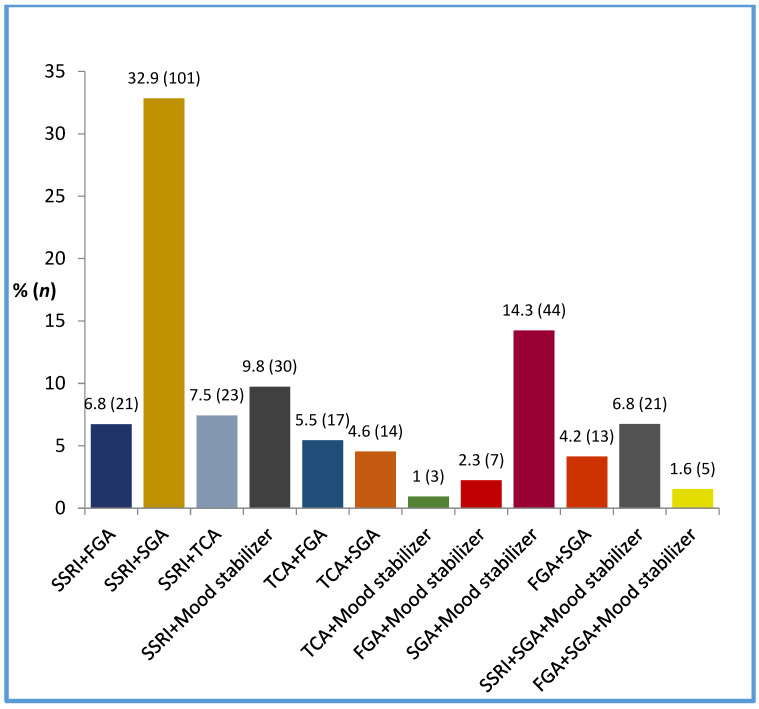
Frequencies of psychotropic classes combinations (*n* = 307).

Among psychotropic drug combinations, the most frequent was SSRIs with SGAs (32.9%), followed by SGAs with mood stabilizers (14.3%) and SSRIs with mood stabilizers (9.8%). A triple combination of SSRIs, SGAs and mood stabilizers occurred in 6.8% of cases (Figure 3).

### 3.3. ECG Changes Caused by Psychotropic Drugs

The RR interval was significantly shorter among patients on TCAs versus those not receiving TCAs and among patients on polytherapy versus those on monotherapy (*p* < 0.05 for both comparisons) after adjusting for confounding factors such as age, gender, BMI, smoking, caffeine intake, presence of diabetes mellitus, cardiovascular disease and medications known to produce heart rate changes (Table 3).

The Kruskal-Wallis Test was conducted to examine the effect of the most common psychotropic combination, SSRIs and SGAs, in comparison to either SSRIs or SGAs or none of these medications on RR, PR, TpTe and TpTe/QT intervals (Table 4). The test provided evidence of a difference (*p* < 0.05) between the mean ranks of RR interval of at least one pair of groups. Pairwise Mann-Whitney U tests were carried out for the four pairs of groups for RR interval. There was strong evidence (*p* < 0.01, adjusted for the number of pairs being tested) of a shorter RR interval among patients who received SGAs compared to those who received SSRIs. The median RR interval for patients treated with SGAs was 750 ms compared to 800 ms for those on SSRIs. There was no evidence of a difference between the other pairs. We did not find difference in PR, TpTe and TpTe/QT intervals between the SSRI + SGA combination and either SSRIs or SGAs or none of these medications.

Table 5 presents correlations of RR, PR, TpTe intervals and TpTe/QT with patient age, BMI and number of psychotropic drugs. No correlations were found in the quetiapine and mood stabilizers groups. Positive correlations were found between the PR interval and age in patients treated with SGAs (r = 0.282; *p* = 0.001), SSRIs (r = 0.247; *p* = 0.001), citalopram (r = 0.330; *p* = 0.001), polytherapy (r = 0.236; *p* = 0.002) and in the total sample (r = 0.252; *p* = 0.001). Inverse correlations were found between the RR interval and the number of psychotropic medications among patients treated with SSRIs (r = −0.178; *p* = 0.007) and in the whole study sample (r = −0.174; *p* = 0.002). None of the ECG parameters correlated with BMI.

We found that one patient (0.3%) had premature ventricular contractions (PVCs) and another one (0.3%) had biphasic T wave. The patient with PVC was a 55-year-old female, overweight (BMI 28.9), smoker, daily caffeine intake 3 cups of coffee, no comorbidities, main psychiatric diagnosis bipolar disorder, on quetiapine (400 mg BID) and lamotrigine (25 mg BID), with a heart rate of 106 BPM. The patient with biphasic T wave was a 38-year-old male, smoker, daily caffeine intake 8 cups of coffee, type 2 DM, main psychiatric diagnosis bipolar disorder, on fluoxetine (20 mg TID), haloperidol (5 mg BID), olanzapine (10 mg QD), alprazolam (0.5 mg BID) and procyclidine (5 mg BID); his heart rate was 99 BPM. No QTc prolongation was determined in both cases.

## 4. Discussion

In this cross-sectional study, we investigated the effect of antipsychotics and other psychotropic drugs on the ECG parameters other than QTc interval in Jordanian outpatients with mental illness, with special emphasis on polypharmacy. Polypharmacy relates to the use of more than one medication, typically with a complex medication dosing schedule. However, there is no adequate evidence to support the safety and efficacy of such treatment regimens [1].

It is well known that high resting heart rate is independently associated with increased risk of cardiovascular mortality [20,21]. FGAs, SGAs and TCAs have been previously associated with a shorter RR interval [22,23,24] due to the presence of α^2^ adrenoceptor- and muscarinic blocking effect [1]. In our study, the RR interval was significantly shorter among patients on TCAs versus those not receiving TCAs. Such effects were previously reported by van Zyl et al. [25]. As expected, patients receiving polytherapy had significantly shorter RR intervals compared to those on monotherapy, as many of the medications prescribed from the antipsychotic and antidepressant classes have antimuscarinic effects.

Prolongation of the TpTe interval [the interval between the peak and the end of the T wave (Tpeak to Tend)], a measure of cardiac transmural dispersion of repolarization, is associated with higher risk of SCD via increased susceptibility to early and late afterdepolarizations [19]. In a cross-sectional study that involved psychiatric inpatients, clozapine increased TpTe [26], and in a randomized double-blind study in schizophrenia patients, sertindole increased TpTe, while quetiapine caused no increase in TpTe [14]. However, the authors of the latter study noted that, although quetiapine did not show worsening of repolarization measures, some individual patients did experience significant worsening of repolarization [14]. We failed to confirm the above data on the effect of quetiapine on TpTe and TpTe/QT ratio.

Positive correlations were found between the PR interval and age consistently in patients treated with SGAs, SSRIs, citalopram, polytherapy and in the total sample (*p* < 0.01 for all). Such correlations are reported for the first time among patients treated by psychotropic agents. Inverse correlations were found between the RR interval and the number of psychotropic medications among patients treated with SSRIs and in the whole study sample (*p* < 0.01 for both), in support of the above data comparing patient on monotherapy vs. those on polytherapy. In contrast, the use of multiple antipsychotics, with or without other psychotropic drugs, was not significantly associated with the presence or number of ECG abnormalities in patients with schizophrenia and schizoaffective disorder in a long-stay inpatient unit [2].

We report one case of PVC and another one of biphasic T wave, both patients treated with antipsychotics (quetiapine and a combination of haloperidol and olanzapine, respectively). The resting heart rate was elevated in both patients, and this is known to be associated with SCD risk. Our findings are consistent with those of a recent study among patients with schizophrenia, where redeemed prescriptions of antipsychotics and antipsychotic polypharmacy were associated with abnormal ECGs [11]. However, the recent observational studies found no significant difference in mortality between patients on antipsychotic polypharmacy vs. those on monotherapy [1].

Study limitations include its cross-sectional observational nature, which does not allow to the drawing of the causation of ECG abnormalities (e.g., mental disease vs. psychotropic medications). We did not consider medication adherence, duration of mental disease or pharmacological treatment. Future large prospective studies are recommended that will address all the above issues, as well as the relation of ECG changes to SCD in patients on antipsychotics and other psychotropic medications.

## 5. Conclusions

Various ECG changes beyond QTc interval prolongation are observed in patients on antipsychotics and other psychotropic medications, and in those on polytherapy. It is recommended to obtain an ECG before starting patients on psychotropic drugs known to produce electrocardiographic changes and their combinations.

## Figures and Tables

**Table 1:** General Characteristics of the Study Participants (*n* = 307).

Patient Characteristic	
Gender, *n* (%)	
Male	141 (45.9)
Female	166 (54.1)
Age, Mean (SD) (range)	36.26 (13.48) (18–81)
BMI, Mean (SD) (range)	26.66 (5.07) (16.40–47.94)
Smoker, ^a^ *n* (%)	113 (36.8)
Alcohol, ^a^ *n* (%)	10 (3.3)
Caffeine, ^a^ *n* (%)	233 (75.9)
Physical activity, ^a^ *n* (%)	61 (19.9)
Psychiatric conditions, *n* (%)	
Anxiety disorders	127 (41.4)
Major depressive disorder	88 (28.7)
Bipolar disorder	53 (17.3)
Schizophrenia	15 (4.9)
Others	24 (7.8)
Non-psychiatric conditions, *n* (%)	
Cardiovascular disease ^b^	34 (5.9)
Hypertension	26 (8.4)
Diabetes mellitus	17 (5.6)
Hypothyroidism	5 (1.6)
Epilepsy	5 (1.6)
Asthma	4 (1.3)
Others	24 (7.8)

^a^ Most days of the week of any amount; ^b^ including hypertension.

**Table 2:** Frequency of individual psychotropic medications.

Name of Class and Medication	Frequency	%
SSRIs	226	100
Citalopram	118	52.2
Fluoxetine	65	28.2
Fluvoxamine	19	8.4
Escitalopram	17	7.5
Sertraline	4	1.8
Paroxetine	3	1.3
SNRIs	3	100
Venlafaxine	2	66.7
Duloxetine	1	33.3
TCAs	35	100
Melitracen	18	51.4
Amitriptyline	13	37.1
Clomipramine	4	11.4
Other antidepressants		
Mirtazapine	8	100
FGAs	33	100
Flupentixol	26	78.8
Haloperidol	3	9.1
Sulpiride	1	3.0
Zuclopentixol	3	9.1
SGAs	158	100
Quetiapine	73	46.2
Olanzapine	44	27.9
Risperidone	29	18.4
Amisulpiride	7	4.4
Aripiprazole	2	1.3
Clozapine	2	1.3
Ziprasidone	1	0.6
Mood stabilizers	69	100
Valproate	27	39.1
Lamotrigine	21	30.4
Lithium	13	18.8
Carbamazepine	8	11.6
Benzodiazepines	24	100
Bromazepam	17	70.8
Alprazolam	5	20.8
Clonazepam	2	8.3

FGAs: first generation antipsychotics, TCAs: tricyclic antidepressants; SGAs: second generation antipsychotics, SNRIs: serotonin and norepinephrine reuptake inhibitors; SSRIs: selective serotonin reuptake inhibitors.

**Table 3:** Effect of the most commonly prescribed psychotropic therapies on RR, PR TpTe intervals and TpTe/QT.

Therapy	QT # (ms) (Uncorrected)	RR (ms)	PR (ms)	TpTe (ms)	TpTe/QT
**FGAs**					
Yes (*n* = 33)	366 ± 23	762 ± 142	152 ± 20	76 ± 16	0.21 ± 0.04
No (*n* = 274)	371 ± 28	806 ± 132	148 ± 19	84 ± 93	0.22 ± 0.23
F		2.97	2.72	0.30	0.01
*p*-value		0.086	0.100	0.58	0.94
**SGAs**					
Yes (*n* = 159)	369 ± 27	783 ± 129	146 ± 18	84 ± 96	0.24 ± 0.24
No (*n* = 148)	373 ± 28	821 ± 1362.66	150 ± 193.19	81 ± 790.01	0.22 ± 0.19
F		0.10	0.08	1.00	0.6
*p*-value					
**Quetiapine**					
Yes (*n* = 73)	368 ± 26	780 ± 125	148 ± 17	97 ± 14	0.26 ± 0.35
No (*n* = 234)	371 ± 28	808 ± 136	148 ± 19	78 ± 64	0.21 ± 0.16
F		0.83	0.02	0.41	0.37
*p*-value		0.36	0.92	0.52	0.54
**SSRIs**					
Yes (*n* = 226)	375 ± 27	814 ± 134	148 ± 18	86 ± 10	0.23 ± 0.25
No (*n* = 81)	359 ± 26	767 ± 127	148 ± 21	72 ± 18	0.20 ± 0.05
F		3.73	0.01	0.07	0.62
*p*-value		0.06	0.94	0.80	0.43
**Citalopram**					
Yes (*n* = 118)	376 ± 29	814 ± 137	150 ± 18	75 ± 19	0.20 ± 0.05
No (*n* = 189)	367 ± 26	794 ± 132	147 ± 19	87 ± 12	24 ± 0.27
F		0.32	1.07	0.19	0.01
*p*-value		0.58	0.30	0.66	0.91
**TCAs**					
Yes (*n* = 35)	369 ± 21	**759 ± 109**	152 ± 13	74 ± 13	0.20 ± 0.05
No (*n* = 271)	371 ± 28	**808 ± 136**	148 ± 19	84 ± 93	0.23 ± 0.23
F		**5.93**	1.81	0.08	0.01
*p*-value		**0.02**	0.18	0.77	0.96
**Mood stabilizers**					
Yes (*n* = 69)	363 ± 27	784 ± 132	149 ± 21	73 ± 16	0.20 ± 0.04
No (*n*= 238)	373 ± 27	807 ± 134	148 ± 18	86 ± 100	0.23 ± 0.25
F		0.89	0.01	0.38	0.01
*p*-value		0.35	0.96	0.54	0.95
**Polytherapy**					
Yes (*n* = 184)	369 ± 27	**782 ± 127**	148 ± 20	81 ± 87	0.23 ± 0.22
No (*n* = 123)	372 ± 28	**830 ± 1397.61**	148 ± 19	83 ± 89	0.22 ± 0.21
F		**0.01**	0.02	0.18	0.01
*p*-value			0.88	0.68	0.96

Significant differences are shown in bold fonts; values in bold indicate significant differences in Quade’s test, with adjusting for: age, gender, BMI, smoking, caffeine intake, presence of diabetes mellitus, cardiovascular disease and presence of medications known to produce either bradycardia (beta-blockers or calcium channel blockers) or tachycardia (beta2-adrenergic agonists or thyroid hormones); # comparisons for QT interval were not conducted as it was used for TpTe/QT calculation only. FGAs: first generation antipsychotics, TCAs: tricyclic antidepressants; SGAs: second generation antipsychotics, SSRIs: selective serotonin reuptake inhibitors.

**Table 4:** The effect of SGA and SSRI combination on RR, PR, TpTe intervals and TpTe/QT.

Interval	Type of Therapy	*n*	Mean Rank	X^2^
RR	None	23	148	5.79
	SSRI	125	170	
	SGA	58	124	
	SSRI + SGA	101	153	
PR	None	23	177	4.87
	SSRI	125	163	
	SGA	58	138	
	SSRI + SGA	101	147	
TpTe	None	23	146	4.47
	SSRI	123	153	
	SGA	55	144	
	SSRI + SGA	100	153	
TpTe/QT	None	23	165	
	SSRI	123	148	
	SGA	55	158	1.57
	SSRI + SGA	100	148	

By Kruskal-Wallis test.

**Table 5:** Correlations of RR, PR TpTe intervals and TpTe/QT with age, BMI and number of psychotropic drugs in the total sample and in patients prescribed the most common psychotropic medications.

		RR (ms)	PR (ms)	TpTe (ms)	TpTe/QT
**SGAs (*n* = 159)**	**Age**				
	Pearson correlation	0.074	**0.282**	−0.006	−0.013
	Sig (2-tailed)	0.360	**<0.001**	0.943	0.870
	**BMI**				
	Pearson correlation	0.005	0.023	−0.069	−0.069
	Sig (2-tailed)	0.952	0.786	0.416	0.415
	***n* of psychotropic medications**				
	Pearson correlation	−0.055	0.021	−0.030	−0.031
	Sig (2-tailed)	0.494	0.795	0.715	0.700
**Quetiapine (*n* = 73)**	**Age**				
	Pearson correlation	0.099	0.169	−0.074	−0.081
	Sig (2-tailed)	0.414	0.161	0.549	0.511
	**BMI**				
	Pearson correlation	0.086	0.050	−0.128	−0.133
	Sig (2-tailed)	0.487	0.688	0.307	0.287
	***n* of psychotropic medications**				
	Pearson correlation	0.030	−0.096	−0.084	−0.083
	Sig (2-tailed)	0.801	0.421	0.485	0.492
**SSRIs** **(*n* = 226)**	**Age**				
	Pearson correlation	0.064	**0.247**	−0.40	0.60
	Sig (2-tailed)	0.344	**<0.001**	0.562	0.374
	**BMI**				
	Pearson correlation	0.033	0.091	−0.097	−0.104
	Sig (2-tailed)	0.638	0.185	0.160	0.133
	***n* of psychotropic medications**				
	Pearson correlation	**−0.178**	0.036	−0.036	−0.030
	Sig (2-tailed)	**0.007**	0.591	0.594	0.654
**Citalopram (*n* = 118)**	**Age**				
	Pearson correlation	0.122	**0.330**	0.043	−0.018
	Sig (2-tailed)	0.91	**<0.001**	0.646	0.849
	**BMI**				
	Pearson correlation	0.105	0.148	0.009	−0.045
	Sig (2-tailed)	0.286	0.122	0.929	0.643
	***n* of psychotropic medications**				
	Pearson correlation	−0.109	0.069	−0.002	0.012
	Sig (2-tailed)	0.240	0.457	0.984	0.895
**Mood stabilizers (*n* = 69)**	**Age**				
	Pearson correlation	0.165	0.086	−0.074	−0.141
	Sig (2-tailed)	0.186	0.491	0.553	0.259
	**BMI**				
	Pearson correlation	−0.138	−0.121	−0.126	−0.056
	Sig (2-tailed)	0.285	0.350	0.328	0.666
	***n* of psychotropic medications**				
	Pearson correlation	−0.096	−0.026	0.153	0.148
	Sig (2-tailed)	0.433	0.831	0.208	0.225
**Polytherapy (*n* = 184)**	**Age**				
	Pearson correlation	0.034	**0.236**	−0.007	−0.14
	Sig (2-tailed)	0.649	**0.002**	0.929	0.851
	**BMI**				
	Pearson correlation	−0.018	0.050	−0.071	−0.070
	Sig (2-tailed)	0.819	0.517	0.367	0.370
	***n* of psychotropic medications**				
	Pearson correlation	−0.072	0.038	−0.064	−0.060
	Sig (2-tailed)	0.334	0.610	0.389	0.419
**Total sample (n = 307)**	**Age**				
	Pearson correlation	0.038	**0.252**	−0.025	−0.035
	Sig (2-tailed)	0.517	**<0.001**	0.666	0.552
	**BMI**				
	Pearson correlation	−0.11	0.053	−0.090	−0.093
	Sig (2-tailed)	0.849	0.374	0.131	0.120
	***n* of psychotropic medications**				
	Pearson correlation	**−0.174**	0.013	−0.020	−0.014
	Sig (2-tailed)	**0.002**	0.823	0.732	0.805

## Data Availability

Data presented in this study are available at the corresponding author on reasonable request.
